# Preamble-Based Adaptive Channel Estimation for IEEE 802.11p †

**DOI:** 10.3390/s19132971

**Published:** 2019-07-05

**Authors:** Joo-Young Choi, Han-Shin Jo, Cheol Mun, Jong-Gwan Yook

**Affiliations:** 1 Department of Electrical and Electronic Engineering, Yonsei University, Seoul 03722, Korea; 2 Department of Electronics and Control Engineering, Hanbat National University, Daejeon 34158, Korea; 3 Department of Electronic Engineering, Korea National University of Transportation, Chungju 27469, Korea

**Keywords:** IEEE 802.11p, Preamble, Adaptive Channel Estimation, Intelligent Transportation Systems, Vehicular Communications

## Abstract

Recently, research into autonomous driving and traffic safety has been drawing a great deal of attention. To realize autonomous driving and solve traffic safety problems, wireless access in vehicular environments (WAVE) technology has been developed, and IEEE 802.11p defines the physical (PHY) layer and medium access control (MAC) layer in the WAVE standard. However, the IEEE 802.11p frame structure, which has low pilot density, makes it difficult to predict the properties of wireless channels in a vehicular environment with high vehicle speeds; thus, the performance of the system is degraded in realistic vehicular environments. The motivation for this paper is to improve the channel estimation and tracking performance without changing the IEEE 802.11p frame structure. Therefore, we propose a channel estimation technique that can perform well over the entire SNR range of values by changing the method of channel estimation accordingly. The proposed scheme selectively uses two channel estimation schemes, each with outstanding performance for either high-SNR or low-SNR signals. To implement this, an adaptation algorithm based on a preamble is proposed. The preamble is a signal known to the transmitter–receiver, so that the receiver can obtain channel estimates without demapping errors, evaluating performance of the channel estimation schemes. Simulation results comparing the proposed method to other schemes demonstrate that the proposed scheme can selectively switch between the two schemes to improve overall performance.

## 1. Introduction

In recent years, traffic congestion and accidents have become important global issues caused by road traffic [1]. To deal with these problems, the Cooperative Intelligent Transport System (C-ITS) is attracting a lot of attention, and the Wireless Access in Vehicular Environments (WAVE) has been developed and researched to support C-ITS [2,3,4,5,6]. The WAVE system is a radio communication system that provides standardized services and interfaces to enable vehicle-to-vehicle (V2V) and vehicle-to-infrastructure (V2I) communications [7,8]. In WAVE, the physical (PHY) and medium-access control (MAC) layers are defined using the IEEE 802.11p and IEEE 802.11p/IEEE 1609.4 standards, respectively [9,10]. This system has the potential to significantly increase road safety, and it is also expected to solve many traffic issues. In practical C-ITS applications, the fairness and reliability of the communications between vehicles using the WAVE system is very important. Fairness problems arising from IEEE 802.11p MAC affects the throughput and packet drop rate; thus, various MAC schemes have been proposed to solve these problems [11]. In terms of reliability, the accuracy of channel estimation plays a critical role in improving the reliability of communications in vehicular environments because it generally affects the system performance, including equalization, demodulation, and decoding performance.

In vehicular environments, wireless channels have a high Doppler shift and large delay spread [12,13,14]. As a result, the channel shows time and frequency selective fading characteristics due to motion and multipath components. The channel variations that occur in vehicular environments affect the transmitted packet; thus, the channel coefficient can significantly change between the start and end of the packet. To adapt to these rapid channel variations, IEEE 802.11p PHY was designed based on IEEE 802.11a PHY [15]. The 802.11p has a bandwidth of 10 MHz which is halved bandwidth of 802.11a and the carrier frequency is 5.9 GHz, higher than 802.11a. Except for the bandwidth and carrier frequency, the 802.11p PHY has the same structure as 802.11a; thus, 802.11p uses four pilot subcarriers in each symbol portion of the packet. However, these pilot subcarriers are insufficient for tracking the channel variations in vehicular environments [12,13]. This leads to poor system performance in realistic vehicular environments. Thus, robust and accurate channel estimation is necessary to obtain excellent communication performance.

### 1.1. Related Work and its Limitations

From this perspective, various channel estimation techniques have been proposed to enhance system performance. They are typically divided into two categories, i.e., with and without changing the IEEE 802.11p standard. With respect to the schemes that change the standard structure [16,17,18,19,20,21], because they can supplement the lack of pilots of IEEE 802.11p, outstanding performance can be obtained compared to approaches that do not modify IEEE 802.11p. However, these methods cause compatibility and interoperability problems with standard equipment.

Because of the problems mentioned above, the majority of channel estimation schemes do not change the standard structure [22,23,24,25,26,27,28,29,30]. In [22], Wiener filter and decision-directed estimation are employed to minimize the mean squared error (MSE). This scheme requires the characteristics of a channel that are difficult to obtain in vehicular environments. In [23,24], an adaptive reduced-rank estimation using subspace selection and an iterative estimation using generalized discrete prolate spheroidal sequences are proposed, respectively. However, both schemes employ the Wiener filter and iterative method for channel estimation, resulting in high computational complexity. In [25], a spectral temporal averaging (STA) method was presented to improve error performance. the STA scheme performs a moving average of the initial channel estimates in the time and frequency domains so as to mitigate noise effects and demapping errors. Therefore, the STA scheme performs well at low signal-to-noise ratio (SNR) regions with strong noise, but an error floor occurs in high-SNR regions. In [26,27], constructed data pilots (CDPs) and time-domain reliable test frequency domain interpolation (TRFI) are, respectively, addressed using the correlation characteristics of channels between adjacent symbols. These schemes obtain excellent performance in high-SNR regions of the signal. However, in low-SNR regions, the correlation characteristics are very poor due to noise, thus degrading performance. In [28], a minimum MSE (MMSE) scheme based on a virtual pilot subcarrier was proposed. In contrast to conventional MMSE which requires prior knowledge of a channel, this scheme employs virtual pilot subcarriers to obtain a covariance matrix; thus, it is less complex. Although the MMSE scheme shows good performance in certain cases, it still performs worse than STA in low-SNR signal regions. To achieve outstanding performance at all SNR levels, two-channel estimation schemes were developed in [29]. First, a modified CDP (MCDP) was proposed that compensates for poor performance using a Markov process in the low-SNR region of the CDP. However, MCDP has a higher error rate than CDP in high-SNR regions; thus, SNR-assisted MCDP (SAMCDP), which selectively uses MCDP and CDP according to a predefined SNR threshold, has been proposed. Because the SNR threshold may change depending on the channel environment, an adaptation method based on the SNR threshold cannot obtain high accuracy. In [30], channel estimation based on self-organization of frequencies was proposed to mitigate the time-varying channel effects. However, this method requires decoding and encoding processes that are equal to the number of symbols in a packet for obtaining the channel estimate, leading to high computational complexity.

### 1.2. Our Motivation and Contribution

The main motivation for this paper is to obtain a satisfactory performance over the entire range of SNR levels by improving the channel estimation and tracking performance (without changing the packet structure), and to comply with the IEEE 802.11p standard. In [31], we have proposed an adaptive channel estimation scheme that selectively uses two channel estimation schemes to improve the error performance for the entire SNR range of interest, and have investigated that the channel estimation performance can be improved by selectively using the better estimation scheme between schemes STA and MMSE. In this paper, we introduce an enhanced scheme of the adaptive channel estimation. The main contributions of this paper are given as follows.

- In our previous study [31], the proposed algorithm lacked a clear rationale. Therefore, in this study, we provide a theoretical analysis of the proposed algorithm. that can verify its validity.
- By using the standard IEEE 802.11p channel model of [32,33], we evaluate the bit error rate (BER) and packet error rate (PER) of the proposed scheme. By adopting the standard IEEE 802.11p channel model, it can provide fair and objective performance assessment of the proposed scheme.
- We show that the proposed scheme can selectively use the STA and MMSE or STA and TRFI channel estimation schemes. This proves that the proposed scheme can be expanded for any combinations of a channel estimation scheme.

### 1.3. Paper Overview

Section 2 outlines the IEEE 802.11p and then introduces the channel models considered in the simulation. Then, the practical channel estimation schemes used by the proposed scheme are introduced in Section 3. Section 4 presents the adaptation algorithm using the long preamble for adaptive channel estimation. The error performance of the simulation results for the proposed method are analyzed with respect to the results of other channel estimation schemes in Section 5. Finally, Section 6 concludes this paper.

## 2. IEEE 802.11p Standard and Channel Model

### 2.1. IEEE 802.11p PHY Layer

**Figure 1:**
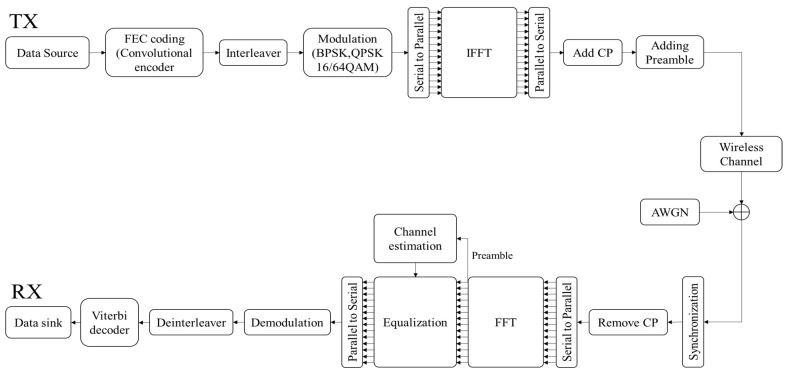
Block diagram of transmitter and receiver in OFDM system.

Orthogonal frequency division multiplexing (OFDM) is employed in the IEEE 802.11p PHY layer. The OFDM transmitter sends several parallel data streams through several orthogonal subcarriers, thereby improving the spectral efficiency and mitigating the severity of multipath fading. The transmitter and receiver in the OFDM system are shown in Figure 1. In the transmitter, a convolutional encoder is first utilized for forward error correction. Puncturing can be used to obtain higher code rate, e.g., 2/3 or 3/4. The coded data is then interleaved to avoid burst errors. Subsequently, the signal is modulated according to the modulation scheme. After that, a 64-point inverse fast Fourier transform (IFFT) is performed with 48 data subcarriers, four pilot subcarriers, and 12 null subcarriers. Finally, the cyclic prefix (CP) and preamble are added. The CP is employed to prevent inter-symbol and inter-carrier interference, and the preamble is primarily employed for synchronization and channel estimation.

In the receiver, assuming perfect timing and frequency synchronization, the CP is first eliminated from the received packet. After the signal-to-parallel (S/P) and fast Fourier transform (FFT) blocks, the preamble and frequency guard band are removed in turn and the channel coefficients are estimated by two long training symbols. IEEE 802.11p uses a 1-tap equalizer, which compensates for the channel fading effect by using estimated channel coefficients. The equalized data symbols are then demodulated according to the modulation scheme. After the decoder and deinterleaver, the demodulated data bits are converted to sink bits. Table 1 represents the specific system parameters defined in IEEE 802.11p.

**Figure 2:**
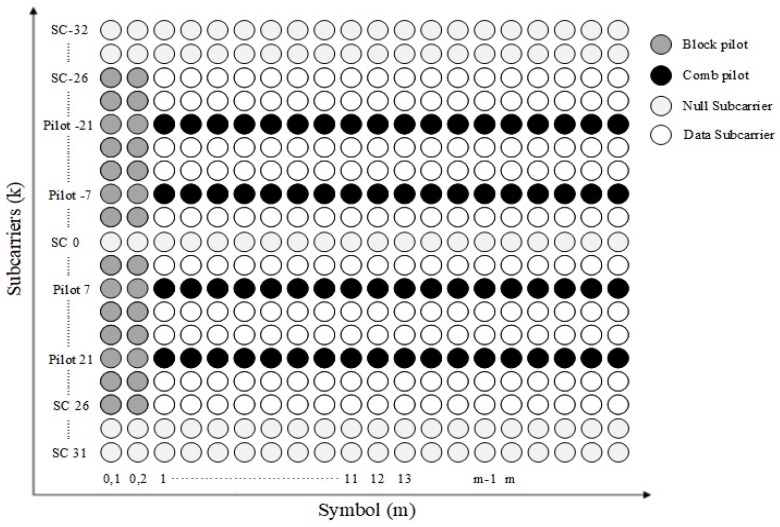
Pilot arrangement of IEEE 802.11p.

The 802.11p PHY layer also defines two types of pilot arrangements, i.e., comb and block types, for channel estimation, as shown in Figure 2. The comb-type arrangement dedicates four uniformly distributed pilot subcarriers in each OFDM symbol whereas the block type dedicates all subcarriers as the pilot except for those dedicated to the null subcarriers. A single OFDM symbol is composed of a total of 64 subcarriers, numbered −32 to 32; 48 data subcarriers are assigned numbers from −26 to 26, not including −21, −7, 0, 7, and 21. The pilot subcarriers are allocated to subcarriers −21, −7, 7, and 21. The remaining indexes, i.e., −32 to −27, 0, and 28 to 31, are used as null subcarriers. IEEE 802.11p uses a block-comb pilot pattern that combines these two types. As shown in Figure 2, two OFDM symbols of block type represent long training symbols in the preamble and the remaining OFDM symbols utilize the comb-type.

In these pilot patterns, the block pilot and comb pilot are suitable for frequency-selective fading and fast-fading, respectively. However, in vehicular environments, the coherence time is typically shorter than the packet duration, and the coherence bandwidth is smaller than the four pilot-subcarrier spacings [12,13]. Therefore, accurate channel estimation is difficult when only the pilots defined in the IEEE 802.11p are employed, which degrades performance.

### 2.2. Channel Model in Vehicular Environments

Generally, a mobile radio channel in vehicular environments is a time-varying channel including multi-path propagation and a large Doppler shift [12,13,14]. Such a channel can be implemented as a tapped delay line model (TDL) which is commonly used for implementing the multi-path channel. The fading of the paths constituting each tap is summed up to obtain Rician or Rayleigh fading, h(t,τ), which varies with the duration of each tap. The impulse response of small fading with time-frequency correlation through Doppler and delay spread is represented as follows.

(1) $$\begin{array}{c} h\left(t,\tau \right)=\sum_{l=1}^{L}\beta_{l}\left(t\right)\delta \left(\tau -\tau_{l}\left(t\right)\right)e^{j2\pi f_{d,l}t}, \end{array}$$

where $\beta_{l}\left(t\right)$, $\tau_{l}\left(t\right)$, and $f_{d,l}$ represent the amplitude, delay, and Doppler shift of the *l*-th path, respectively. As the performance of an actual wireless system depends on the state of the channel, it is very important to analyze the statistical characteristics of the wireless channel; thus, various studies on vehicle to everything (V2X) channels have been conducted [32,33,34,35,36,37,38,39].

In this study, we use the TDL model proposed in [32,33] to consider the Doppler and delay spread in realistic vehicular environments. These channel models are classified into six models according to different scenarios. In the TDL model, each tap has been schematized, including tap power, delay value, and Doppler spectrum. A concise summary of the proposed six-channel model is provided in [32]. Of the six models, we consider only two channel models (as shown in Table 2), i.e., V2V Expressway Oncoming and V2I Urban Canyon, owing to the importance of the real situation in the six models.

## 3. Channel Estimation Schemes

In this section, we describe the practical channel estimation schemes that can be selectively used by the proposed scheme. These channel estimation schemes are compared with the proposed scheme in Section 5.

### 3.1. LS Estimation Scheme

The LS scheme performs channel estimation using predefined long training symbol $X_{0}\left(k\right)$ in frequency domain. Since the preamble is composed of two identical long training symbols, the LS channel estimation is obtained as:

(2) $$H\left(0,k\right)=\frac{Y_{0}\left(1,k\right)+Y_{0}\left(2,k\right)}{2X_{0}\left(k\right)},$$

where $Y_{0}\left(1,k\right)$ and $Y_{0}\left(2,k\right)$ are the frequency-domain received symbols at subcarrier *k*. The channel estimates obtained by (1) are employed for equalizing all the received symbols in the packet; the channel variations in vehicular environments are very fast, so channel estimate H(0,k) is not valid over time. Hence, the LS scheme is difficult to use in vehicular environments.

### 3.2. STA Estimation Scheme

To track rapidly changing channel variations, the STA scheme continuously updates the channel estimates. First, the m-th frequency domain received symbol Y(m,k) is divided using the previous channel estimate H(m−1,k) as follows:

(3) $$\hat{T}\left(m,k\right)=\frac{Y(m,k)}{H(m-1,k)},m=1,\cdots ,M.$$

where H(0,k) is equal to (1).

Then, $\hat{T}\left(m,k\right)$ is demapped to $\hat{X}\left(m,k\right)$ and the demapped symbol is again used to obtain initial channel estimates $\hat{H}\left(m,k\right)$ such that,

(4) $$\hat{H}\left(m,k\right)=\frac{Y(m,k)}{\hat{X}\left(m,k\right)}.$$

Next, to mitigate noise effects and demapping errors in $\hat{H}\left(m,k\right)$, the moving average of the data subcarriers in the frequency-domain received symbols is implemented in the frequency and time domains as

(5) $$\tilde{H}\left(m,k\right)=\sum_{\lambda =-\beta }^{\lambda =\beta }\omega_{\lambda }\hat{H}\left(m,k+\lambda \right)$$

(6) $$H\left(m,k\right)=\left(1-\frac{1}{\alpha }\right)H\left(m-1,k\right)+\frac{1}{\alpha }\tilde{H}\left(m,k\right),$$

where $\omega_{\lambda }$ is a set of weighting coefficients defined by 1/(2β+1), and parameter β and α are the moving average parameters in frequency and time domains, respectively. These parameters should be adaptively varied depending on the channel conditions, but it is very difficult task. Therefore, STA uses fixed values that show optimal performance, i.e., β=α=2 [25].

Using the moving average, STA can effectively reduce the demapping errors induced by noise. However, in high-SNR regions, demapping errors mainly occur due to fading; thus, channel estimation error is generated by the moving average, thereby, causing an error floor.

### 3.3. TRFI Estimation Scheme

The TRFI scheme improves performance of the channel estimation by using the fact that adjacent subcarriers and symbols have high frequency and time correlation properties, respectively. First, the TRFI computes (1), (2) and (3) above. Channel estimate $\hat{H}\left(m,k\right)$ is then employed to equalize the previous received symbol of frequency domain, Y(m−1,k), as

(7) $${\hat{T}}^{\prime }\left(m-1,k\right)=\frac{Y(m-1,k)}{\hat{H}\left(m,k\right)}.$$

Then, Y(m−1,k) is equalized by H(m−1,k), which is previous symbol’s channel estimates.

(8) $${\hat{T}}^{\prime \prime }\left(m-1,k\right)=\frac{Y(m-1,k)}{H(m-1,k)}.$$

After that, ${\hat{T}}^{\prime }\left(m-1,k\right)$ and ${\hat{T}}^{\prime \prime }\left(m-1,k\right)$ are respectively demapped to ${\hat{X}}^{\prime }\left(m-1,k\right)$ and ${\hat{X}}^{\prime \prime }\left(m-1,k\right)$ according to the modulation scheme, for use in the time reliability test.

In the time reliability test, if ${\hat{T}}^{\prime }\left(m-1,k\right)$ and ${\hat{T}}^{\prime \prime }\left(m-1,k\right)$ are demapped to the same symbol, the initial channel estimate $\hat{H}\left(m,k\right)$ is determined to be the lastest channel estimate. Otherwise, frequency interpolation is performed using the channel estimates that have passed the time reliability test to obtain the most recent channel estimates.

The TRFI scheme relies on the precision of the channel estimates that pass the time reliability test. This scheme performs well in high-SNR regions; however, it cannot exactly track the channel variations in low-SNR regions, which reduces the accuracy of the channel estimates; thus, the TRFI scheme perform poorly in low-SNR regions.

### 3.4. MMSE Estimation Using a Virtual Pilot Subcarrier Scheme

The MMSE is a technique that designates arbitrary virtual pilot subcarriers in frequency-domain-received symbols and uses them to enhance the accuracy of channel estimation. The virtual pilots are preliminarily designated data subcarriers, which are uniformly allocated to maintain the correlation with the subcarriers, i.e., the data and comb pilot subcarriers. The MMSE scheme employs virtual pilots to obtain channel covariance matrices and performs frequency-domain channel estimation using them.

In the MMSE scheme, the initial channel estimates of the m-th OFDM symbol obtained by (3) are first converted into column as

(9) $$\begin{array}{c} {\hat{H}}_{m}=[\hat{H}\left(m,-26\right),\hat{H}\left(m,-25\right),\hat{H}\left(m,k\right)\\ \cdots \;\hat{H}{\left(m,26\right)]}^{T},\;k\in S_{d},\;S_{p} \end{array}$$

where $S_{d}$ and $S_{p}$ represents the index set of data and comb pilot subcarriers mentioned Section 2.1, respectively.

The channel estimates of the comb and virtual pilots are then represented in vector notation

(10) $$\begin{array}{c} P_{m}=[\hat{H}\left(m,-21\right),\hat{H}\left(m,-7\right),\hat{H}\left(m,k\right)\\ \cdots \;\hat{H}{\left(m,21\right)]}^{T},\;k\in S_{p},\;S_{vp} \end{array}$$

where $S_{vp}$ denotes index set of virtual pilots. A total of virtual pilot can be changed as 4, 12, or 48 in an OFDM symbol. The last channel estimate vector is then obtained by

(11) $$H_{MMSE}^{m}=R_{\hat{H}P}^{m}{\left(R_{PP}^{m}+\sigma_{m}^{2}I\right)}^{-1}P_{m},$$

where $R_{\hat{H}P}^{m}$ and $R_{PP}^{m}$ represent the cross-correlation and auto-correlation matrix, respectively. These matrices are expressed as

(12) $$R_{\hat{H}P}^{m}=\frac{1}{m}\sum_{j=1}^{m}{\hat{H}}_{j}P_{j}^{H}$$

(13) $$R_{PP}^{m}=\frac{1}{m}\sum_{j=1}^{m}P_{j}P_{j}^{H},$$

where I denotes the unit matrix of $N_{p}\times N_{p}$. $\sigma_{m}^{2}$ is the average noise power of the *m*th received symbol, and $N_{p}$ is the total number of comb and virtual pilots in each OFDM symbol.

The MMSE scheme uses these correlation matrices to enhance the channel estimation performance. In addition, unlike conventional MMSE, prior knowledge of a channel, i.e., the root mean square (RMS) delay spread and doppler frequency, are not required, and the complexity of the proposed scheme can hence be reduced. This scheme performs well in certain cases, but it still cannot provide outstanding performance compared to STA in low-SNR regions.

## 4. Adaptive Channel Estimation Scheme Based on Preamble

**Figure 3:**
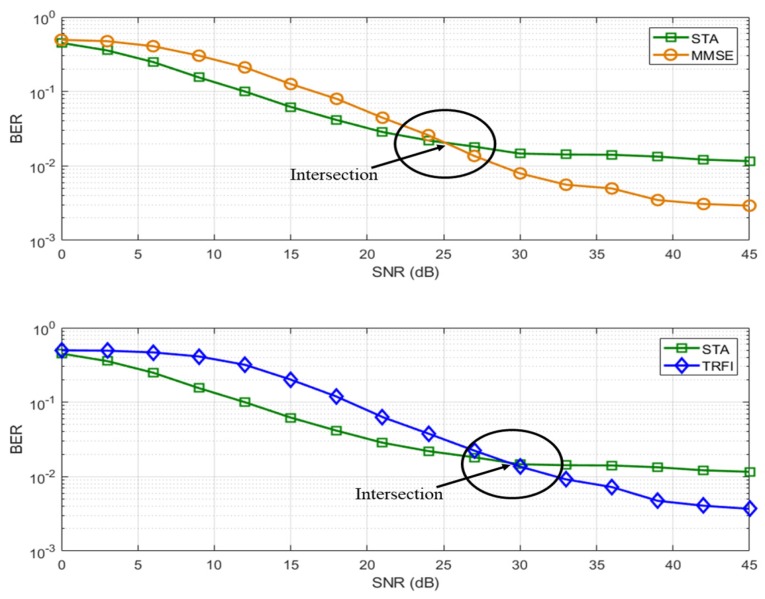
BER performance of QPSK 1/2 in V2I Urban Canyon.

As mentioned above, the number of pilots defined in the IEEE 802.11p is not sufficient to track the channel variations for high-speed vehicle environments. For this reason, various channel estimation techniques have been proposed, but satisfactory performance cannot be obtained over the entire range of SNR levels. Figure 3 shows the BER performance of each channel estimation scheme obtained by using the IEEE 802.11p link level simulator and by adopting V2I channel model in urban canyon scenario mentioned in Section 2.2. In Figure 3, STA, MMSE and TRFI have different SNR region with excellent performance; thus, if a technique that exhibits excellent performance in the corresponding SNR region can be appropriately used, this leads to a performance improvement. In addition, STA and MMSE or STA and TRFI create an intersection, which means that two techniques can be used appropriately based on the intersection. Therefore, in this paper, we propose an adaptive channel estimation technique to obtain outstanding performance regardless of the level of SNR in a signal region. The proposed scheme uses one of two channel estimation techniques that exhibit excellent performance at either high- or low-SNRs.

The adaptive channel estimation scheme chooses the channel estimation technique using a preamble. The preamble is a known bit sequence to both transmitter and receiver in order to estimate channel state information (CSI), and the LS technique is widely used in this preamble-based channel estimation due to its simplicity. LS channel estimate can be obtained as follows:

(14) $$\begin{array}{c} H_{LS}\left(k\right)=\frac{Y(k)}{X(k)}=H_{p}\left(k\right)+\frac{Z(k)}{X(k)}, \end{array}$$

where $H_{p}\left(k\right)$ is the perfect CSI and Z(k) is an additive white gaussian noise (AWGN) with zero mean and variance $\sigma_{z}^{2}$, i.e., $N(0,\sigma_{z}^{2})$. Because $H_{LS}\left(k\right)$ is still influenced by noise component, $H_{i}\left(k\right)$ can be obtained by using a candidate channel estimation technique to reduce the noise effect.

To compare the performance of candidate channel estimation schemes, modified MSE is defined as

(15) $${\tilde{\delta }}_{i}=\frac{1}{N}\sum_{k=-26}^{26}{\left|H_{LS}\left(k\right)-H_{i}\left(k\right)\right|}^{2}.$$

Under the assumption that $H_{i}\left(k\right)\approx H_{p}\left(k\right)$, modified MSE is approximated as follows:

(16) $$\begin{array}{c} {\tilde{\delta }}_{i}\approx \frac{1}{N}\sum_{k=-26}^{26}{\left|H_{LS}\left(k\right)-H_{p}\left(k\right)\right|}^{2}. \end{array}$$

The MSE between the LS channel estimate and the perfect CSI is an inversely proportional average SNR [40]

(17) $$\begin{array}{cc} \delta_{LS} & =\frac{1}{N}\sum_{k=-26}^{26}{\left(H_{LS}\left(k\right)-H_{p}\left(k\right)\right)}^{H}\left(H_{LS}\left(k\right)-H_{p}\left(k\right)\right)\\ & =\frac{1}{N}\sum_{k=-26}^{26}{\left(\frac{Y(k)}{X(k)}-H_{p}\left(k\right)\right)}^{H}\left(\frac{Y(k)}{X(k)}-H_{p}\left(k\right)\right)\\ & =\frac{1}{N}\sum_{k=-26}^{26}{\left(\frac{Z(k)}{X(k)}\right)}^{H}\left(\frac{Z(k)}{X(k)}\right)\\ & =\frac{1}{N}\sum_{k=-26}^{26}\frac{Z^{H}\left(k\right)Z\left(k\right)}{X^{H}\left(k\right)X\left(k\right)}\\ & =\frac{\sigma_{z}^{2}}{\sigma_{x}^{2}}\\ & =\frac{1}{\bar{\gamma }}, \end{array}$$

where $\bar{\gamma }$ is average SNR. Therefore, the better channel estimation technique between the candidate schemes is closer to the perfect CSI, and the modified MSE ${\tilde{\delta }}_{i}$ approaches $1/\bar{\gamma }$. Finally, adaptation algorithm, $\zeta_{i}$ is:

(18) $$\zeta_{i}=\left|\frac{1}{\bar{\gamma }}-{\tilde{\delta }}_{i}\right|.$$

The proposed scheme compares the resulting value obtained by performing the adaptation algorithm on the candidate schemes, and then selects a channel estimation scheme with the smallest resulting value.

**Figure 4:**
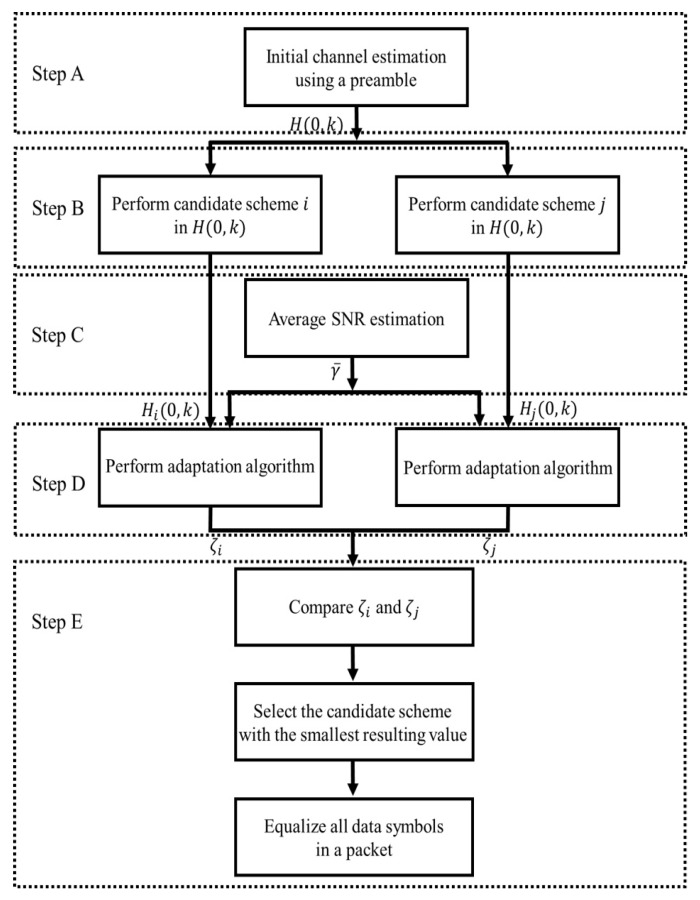
Flowchart of the proposed scheme.

Figure 4 represents the flowchart of the proposed scheme. As shown in Figure 4, the proposed technique consists of the following five steps. (For ease of technical explanation, specific cases, i.e., switching between the STA and MMSE schemes or between the STA and TRFI schemes, are explained by example.)

### 4.1. LS Estimation

The channel estimate of the long training synbols is first obtained through LS estimation using (1).

### 4.2. Performing Channel Estimation

In this step, both channel estimation schemes are separately performed on the channel estimate obtained in the initial step. As mentioned above, this step is described for each specific case. When switching between STA and MMSE, the methods described in Section 3.2 and Section 3.4 are used as follows:

(19) $$H_{STA}\left(0,k\right)=\sum_{\lambda =-\beta }^{\lambda =\beta }\omega_{\lambda }H\left(0,k+\lambda \right).$$

(20) $$H_{MMSE}^{0}=R_{HP}^{0}{\left(R_{PP}^{0}+\sigma_{0}^{2}I\right)}^{-1}P_{0},$$

where parameter β is usually set two in order to obtain best performance [25]. For switching between STA and TRFI, the STA channel estimates are obtained using (18). In the TRFI case, assuming that all channel estimates have passed the time reliability test, the TRFI channel estimate is set to the that of the long preamble H(0,k). Hence, the TRFI channel estimate is defined by

(21) $$\begin{array}{cc} H_{TRFI}\left(0,k\right) & ≜H_{LS}\left(0,k\right)\\ & =\frac{1}{2}\left(\frac{Y(1,k)+Y(2,k)}{2X(k)}\right). \end{array}$$

This channel estimate is used to calculate the adaptation algorithm, and details are covered in Section 4.4.

### 4.3. Estimation of Average SNR

In order to implement the adaptation algorithm, a process for estimating the average SNR is needed. It is very difficult to obtain reliable SNR values in mobile systems; thus, various SNR estimation methods have been proposed to address this problem [41,42,43,44]. In [44], the preamble is employed to estimate the average SNR. This technique has the advantages of being applicable to OFDM-based systems and shows high accuracy [29].

In this paper, we assume that the average SNR estimation is perfect to verify the adaptation algorithm.

### 4.4. Calculation of Adaptation Algorithm

To select the best channel estimation technique, the adaptation algorithm is calculated for each channel estimation technique using the channel estimates and average SNR obtained from the prior steps. The adaptation algorithm derived above according to each channel estimation is expressed as follows.

(22) $$\zeta_{STA}=\left|\frac{1}{\bar{\gamma }}-{\tilde{\delta }}_{STA}\right|$$

(23) $$\zeta_{MMSE}=\left|\frac{1}{\bar{\gamma }}-{\tilde{\delta }}_{MMSE}\right|$$

(24) $$\zeta_{TRFI}=\left|\frac{1}{\bar{\gamma }}-{\tilde{\delta }}_{TRFI}\right|.$$

When switching between STA and MMSE, (21) and (22) are compared, and when switching between STA and TRFI, (21) and (23) are compared.

### 4.5. Selection of Channel Estimation

**Figure 5:**
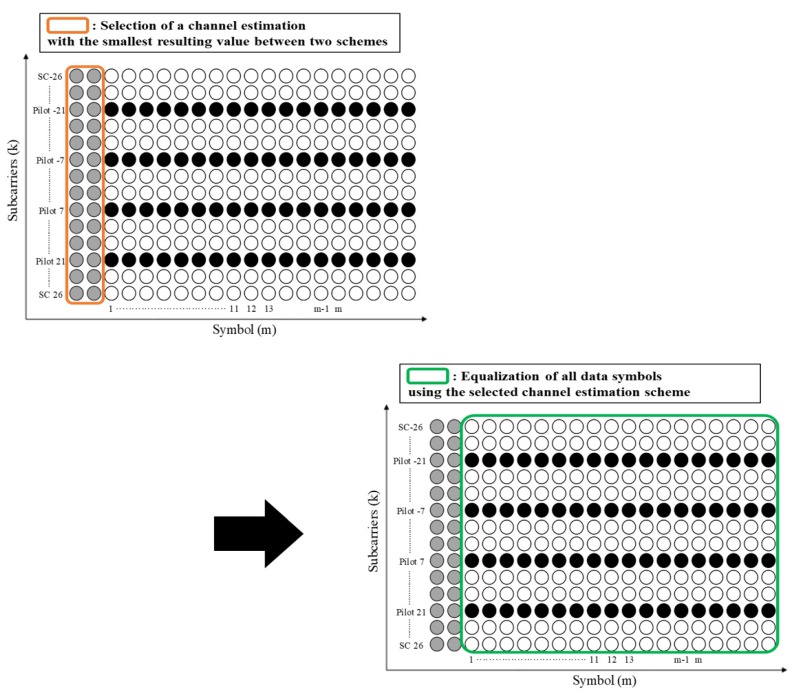
The last step of the proposed scheme.

The key of the proposed scheme is to selectively use a channel estimation scheme that minimizes adaptation algorithm. Therefore, the last step compares the adaptation algorithm of each channel estimation scheme and determines the channel estimation scheme with the smallest resulting value. Comparing the STA to MMSE, it is found that the STA is more efficient than the MMSE at attenuating noise; thus, the STA has a resulting value less than MMSE in low-SNR regions. As a results, the probability that STA is selected increases in low-SNR signal regions. In contrast, as the SNR increases, the effects of noise decreases, and the STA causes an error floor because of the degraded channel estimation accuracy. Therefore, the resulting value of the STA increases as a result of this error floor in high-SNR regions. This leads to an increase in the probability that the STA is not selected. However, because MMSE exhibits excellent channel performance in high-SNR regions, they have a smaller resulting value as the SNR increases than STA, i.e., MMSE have a high probability of being selected. Comparing the STA to TRFI, since the channel estimate of TRFI is set to H(0,k), the modified MSE of TRFI is zero; thus, the adaptation algorithm depends on $1/\bar{\gamma }$. The STA causes an error floor in high-SNR regions, but $1/\bar{\gamma }$ decreases as the SNR increases. Therefore, TRFI is selected after the point where error floor occurs. Figure 5 shows that the channel estimation scheme selected through the last step is employed to equalize all data symbols in the packet.

## 5. Simulation Results

**Figure 6:**
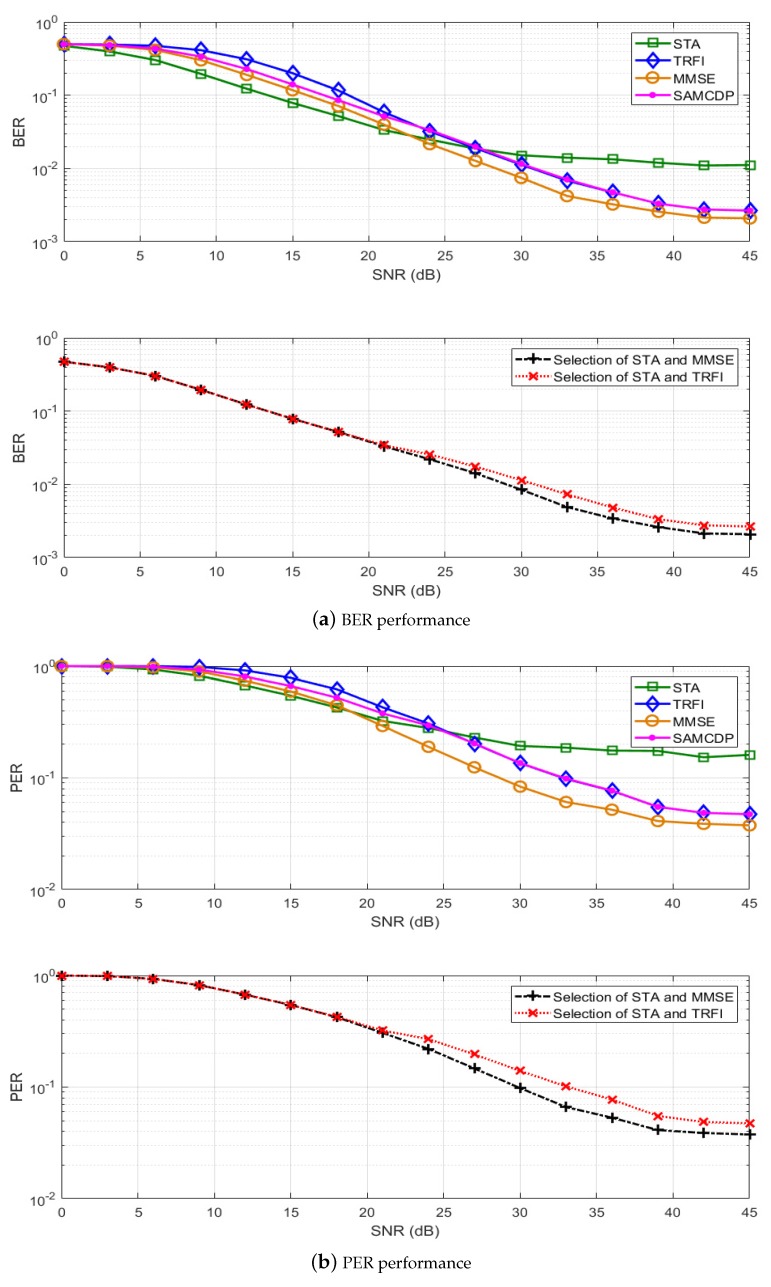
Performance analysis of STA, TRFI, MMSE, SAMCDP, and the proposed scheme (V2V Expressway Oncoming, QPSK 1/2, 50 symbols).

**Figure 7:**
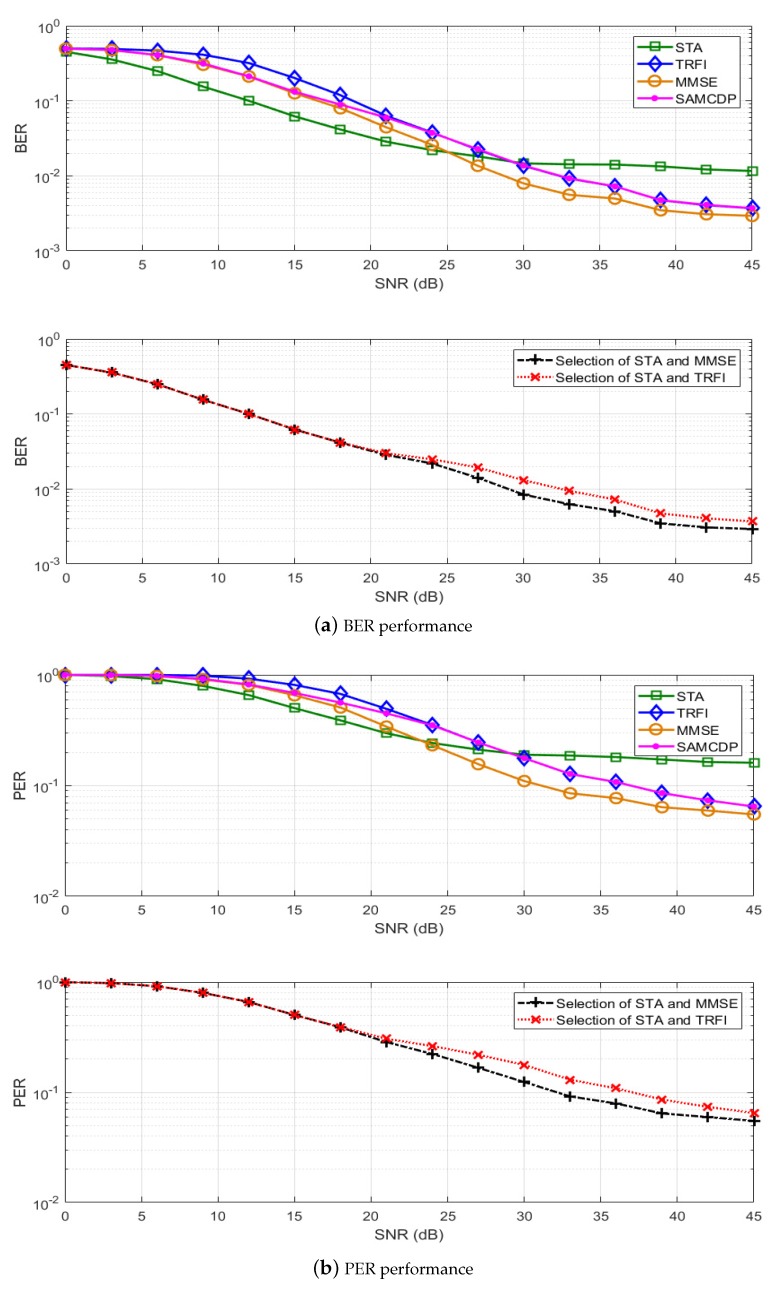
Performance analysis of STA, TRFI, MMSE, SAMMCDP, and the proposed scheme (V2I Urban Canyon, QPSK 1/2, 50 symbols).

**Figure 8:**
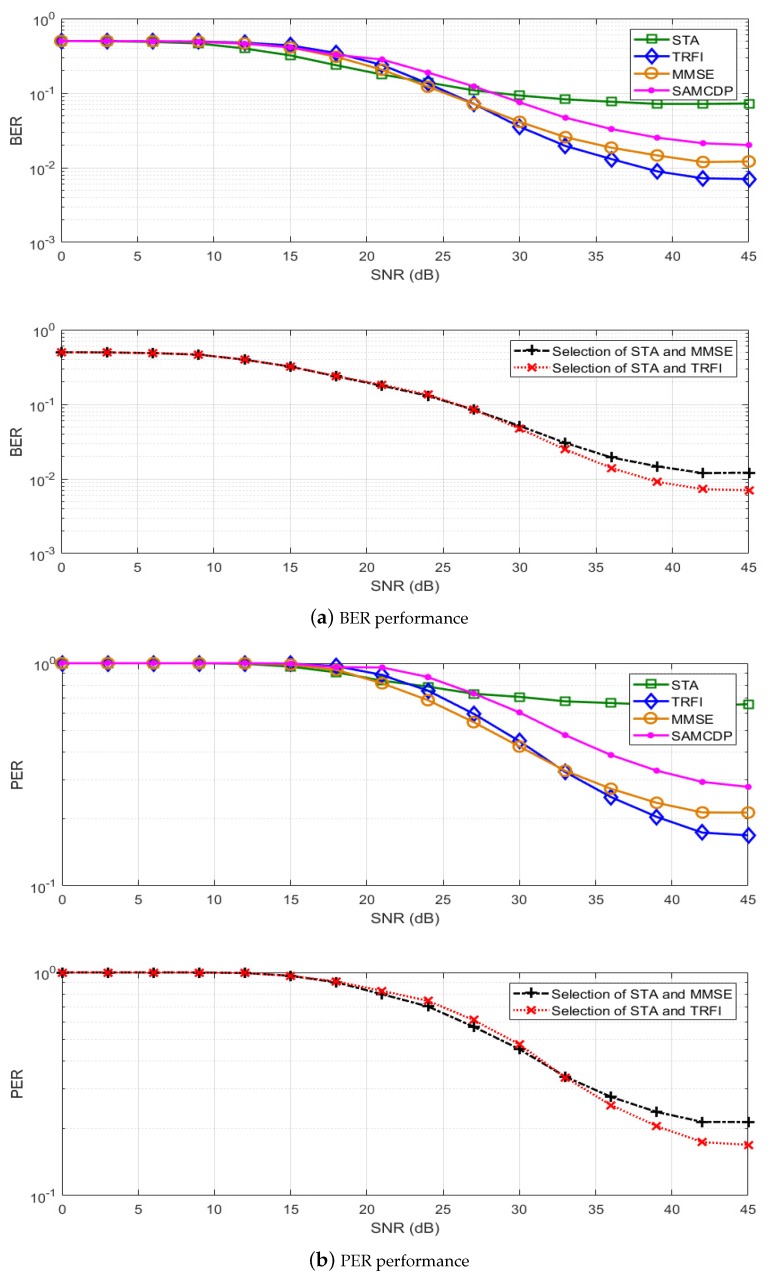
Performance analysis of STA, TRFI, MMSE, SAMMCDP, and the proposed scheme (V2V Expressway Oncoming, 16QAM 1/2, 50 symbols).

**Figure 9:**
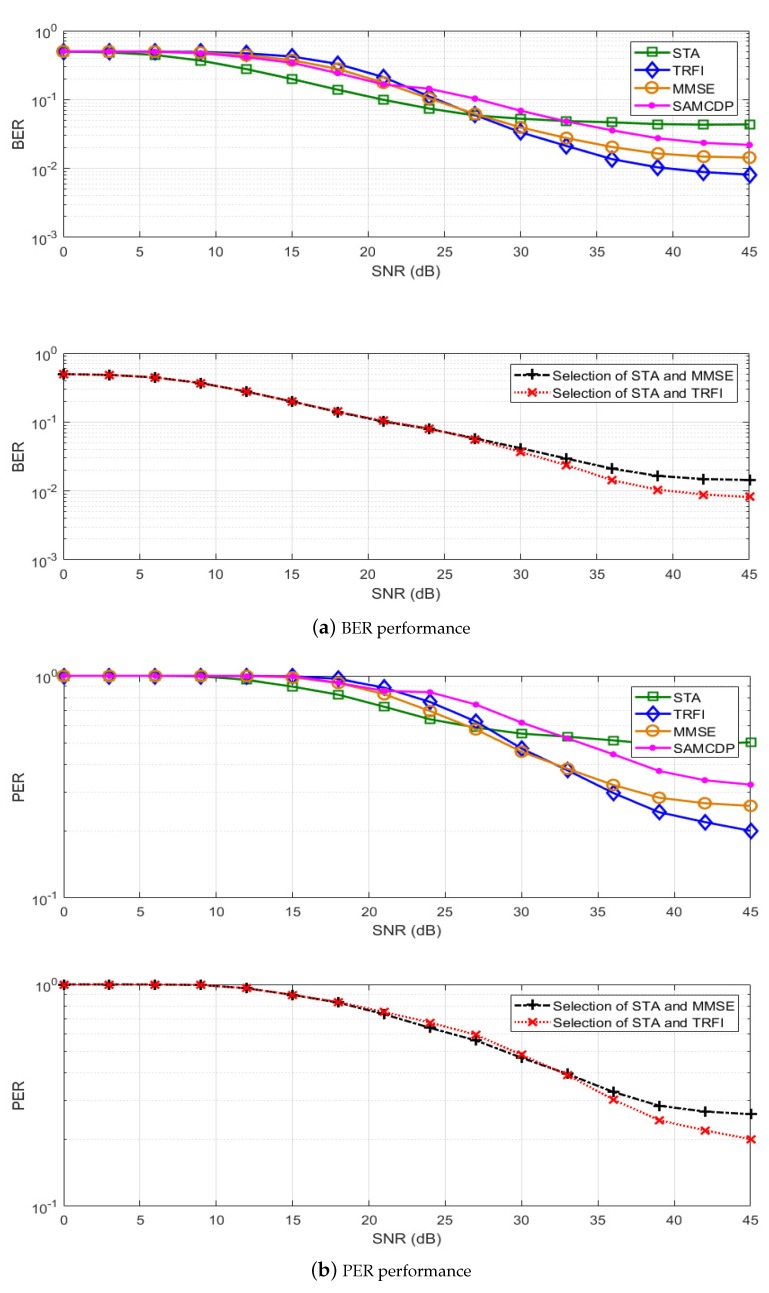
Performance analysis of STA, TRFI, MMSE, SAMMCDP, and the proposed scheme (V2I Urban Canyon, 16QAM 1/2, 50 symbols).

In this section, we present a performance analysis of our proposed scheme. By using the IEEE 802.11p link level simulator, BER and PER simulations have been performed on LS, STA, TRFI, MMSE, SAMCDP, and two versions of the proposed scheme, i.e., switching between STA and MMSE or between STA and TRFI. The IEEE 802.11p link-level simulator was designed to comply with the transmitters and receivers in OFDM systems of IEEE 802.11p (as shown in Figure 1) by using MATLAB. The simulations are performed by following the system parameters in Table 1 and are conducted on V2V and V2I channel models in Table 2. The modulation schemes employ QPSK (Figure 6 and Figure 7) and 16QAM (Figure 8 and Figure 9) with a code rate of 1/2 and the packet is composed of 50 OFDM symbols. The performance of STA depends on the moving average parameters α and β, and that of MMSE depends on the number of virtual pilots. Therefore, to obtain the maximum performance in the STA and MMSE, α and β are set to 2 and 48 virtual pilots are used, respectively.

Figure 6 and Figure 8 present the BER and PER for STA, TRFI, MMSE, SAMCDP, and the proposed scheme in the V2V channel environment, whereas Figure 7 and Figure 9 show the BER and PER of these methods in the V2I channel environment. The left side represents the BER and the right side shows the PER.

The results for the V2V channel model (Figure 6 and Figure 8) reveal that the STA performs well in low-SNR regions, but has an error floor in high-SNR regions. In contrast to STA, MMSE and TRFI performs better only in high-SNR regions. In addition, SAMCDP has lower performance than the channel estimation schemes which show good performance in the high- or low-SNR regions. The proposed scheme provides the performance of STA in low-SNR signal regions and the performance of the corresponding scheme in high-SNR signal regions. Our scheme compared to MMSE, TRFI, and SAMCDP can achieve a performance advantage of about 3–6 dB at a BER for QPSK of 7 × $10^{-2}$ and provide a gain about 2–5 dB at a PER for that of 5 × $10^{-1}$. When STA and MMSE are used for switching, the proposed scheme over TRFI and SAMCDP offers the gain of about 3 dB at a BER of 7 × $10^{-3}$ and provide the advantage about 7 dB at a PER of 8 × $10^{-2}$. In particular, we can see that the error flow of STA, which occurred in high-SNR regions, has been solved in the proposed scheme. In both BER and PER for 16QAM, as with QPSK, the proposed scheme provides the performance of two different schemes depending on the SNRs, and show the performance improvement compared to the other schemes. These results show that in both QPSK and 16QAM, this scheme provides the performance gain over the entire SNR range, as well as the ability to use various channel estimation schemes through proper adaptation algorithm.

For the V2I channel model, Figure 7 and Figure 9 show that the performance of the channel estimation scheme behaves similarly to the performance in the V2V channel model. The STA shows good performance in low-SNR regions, whereas TRFI and MMSE perform very well in the high-SNR regions. The SAMCDP performs worse than STA in the low-SNR regions and worse than TRFI and MMSE in the high-SNR regions. The proposed scheme has the performance of the STA in the low-SNR regions and the performance of the TRFI or MMSE scheme in high-SNR regions. Figure 7a,b shows that the proposed scheme compared MMSE, TRFI and SAMCDP offers about 4–7 dB gain at a BER of 4 × $10^{-2}$ and can achieve about 2–6 dB gain at a PER of about 3 × $10^{-1}$, respectively. In case of switching STA and MMSE, our scheme provides about 6 dB at a BER of 5 × $10^{-3}$ and at a PER of 8 × $10^{-2}$ compared to TRFI and SAMCDP. Figure 9 shows that the proposed scheme has the same trend as QPSK in the BER and PER performances for 16QAM and improves performance over the entire SNRs. These simulation results verify the fact that the proposed scheme is not affected by the channel environment.

## 6. Conclusions

In this paper, we reviewed the IEEE 802.11p standard and described the problems of this standard in time-varying channels. IEEE 802.11p cannot track rapidly changing channel variations because of a lack of pilots for channel estimation. To overcome the channel estimation problems arising from this limitation, various channel estimation techniques that do not need to change the standard have been proposed. However, none of the current channel estimation schemes provide satisfactory performance over the entire range of SNR levels. Therefore, we have proposed an adaptive channel estimation scheme that selectively uses one of two channel estimation schemes with good performance in their corresponding SNR region to obtain outstanding performance over the entire SNR range. The performance for two channel models has been analyzed, and the simulation results demonstrate that the proposed scheme can selectively use two channel estimation techniques regardless of the environment. Nevertheless, the PER of the proposed scheme does not decrease below $10^{-1}$ in 16QAM; thus, we plan to continue our research to increase the performance of high-order modulation.

## Figures and Tables

**Table 1:** IEEE 802.11p system parameters.

Parameter	Value
Bandwidth	10 MHz
Bit rate	3, 4.5, 6, 9, 12, 18, 24, 27 Mbps
Modulation schemes	BPSK, QPSK, 16QAM, 64QAM
Code rate	1/2, 2/3, 3/4
Data subcarriers	48
Pilot subcarriers	4
Total subcarriers	64
FFT period	6.4 μs
CP duration	1.6 μs
Symbol duration	8.0 μs
Subcarrier spacing	0.15625 MHz

**Table 2:** Channel model for simulation.

Scenario	Distance between TX & RX (m)	Velocity (km/h)	Doppler Shift (Hz)	Maximum Excess Delay (μ*s*)
V2V Expressway Oncoming	300–400	104	1000–1200	0.3
V2I Urban Canyon	100	32–48	300	0.5
